# Efficacy and safety of Buzhong Yiqi decoction combined with western medicine in the treatment of myasthenia gravis: A protocol for systematic review and meta-analysis

**DOI:** 10.1097/MD.0000000000024807

**Published:** 2021-03-19

**Authors:** Gang Zong, Shiliang Liu, Zunling Chen, Yulei Hu

**Affiliations:** a Jiaozhou Central Hospital of Qingdao, Jiaozhou; b Community Health Service Center, Qingdao, Shandong Province, China.

**Keywords:** Buzhong Yiqi decoction, myasthenia gravis, protocol, systematic review, Western medicine

## Abstract

**Background:**

Myasthenia gravis is a common autoimmune disease in clinic. Although there are various ways and drugs for the treatment of myasthenia gravis in Western medicine, there are still a variety of adverse reactions. Studies have shown that Buzhong Yiqi decoction combined with Western medicine has a certain efficacy in the treatment of myasthenia gravis, but there is a lack of evidence-based medicine. The research carried out in this scheme is to systematically evaluate the efficacy and safety of Buzhong Yiqi decoction combined with Western medicine in the treatment of myasthenia gravis, and to provide reliable evidence for guiding clinical practice.

**Methods:**

English databases (the Cochrane Library, PubMed, Web of Science, Embase) and Chinese databases (China Biomedical Database, China Science and Technology Journal Database, China National Knowledge Infrastructure, Wanfang) will be searched by computer. In addition, Baidu Academic and Chinese Clinical Trial Registration Center will be searched manually. A randomized controlled clinical trial of Buzhong Yiqi decoction combined with Western medicine in the treatment of myasthenia gravis will be conducted from the establishment of the database to December 2020. The 2 researchers independently carry out data extraction and literature quality evaluation on the quality of the included study, and meta-analysis of the included literature will be carried out by using RevMan 5.3 software.

**Results:**

This study will evaluate the efficacy and safety of Buzhong Yiqi decoction combined with Western medicine in the treatment of myasthenia gravis by Quantitive MGscore, the number of Tregs cells and the content of anti-acetylcholine receptor antibody (AchR-Ab).

**Conclusion:**

This study will provide reliable evidence-based evidence for the clinical application of Buzhong Yiqi decoction combined with Western medicine in the treatment of myasthenia gravis.

**Ethics and dissemination:**

Private information from individuals will not be published. This systematic review also does not involve endangering participant rights. Ethical approval was not required. The results may be published in a peer-reviewed journal or disseminated at relevant conferences.

**OSF Registration number:**

DOI 10.17605/OSF.IO/MXUPK.

## 1 Introduction

Myasthenia gravis (MG) is a destructive acquired autoimmune disease characterized by skeletal myasthenia gravis and fatigue.^[1,2]^ These symptoms can affect oral muscles, lead to dysarthria, and affect about 60% of patients with disease progression.^[3]^ This disease is very common, the prevalence rate is about 1/10,000, the prevalence rate of women is about twice as high as that of men.^[4]^ Ocular myasthenia gravis is the most common phenotype of MG, but it is difficult to predict which cases will continue to develop into systemic MG with ocular symptoms.^[5,6]^ In the early onset of MG, the thymus appears functional and morphological abnormalities, such as B cell infiltration leading to follicular hyperplasia and the production of anti-acetylcholine antibodies. Anti-acetylcholine (Ach) antibodies specifically target postsynaptic acetylcholine receptor (AchR) to cause MG, which leads to the evolution of typical symptoms of the disease, from mild diplopia, muscle fatigue, and repeated exercise, to severe effects on respiratory muscles.^[7,8]^ The thymus is where these antibodies are produced, and abnormalities in the thymus and the abnormal production of these antibodies are associated with MG. Therefore, thymectomy is a common method for the treatment of MG.^[9]^ Although thymectomy improves clinical results, many patients cannot achieve completely stable remission without additional immunosuppressive therapy.^[10]^ In addition, steroids can also be used for immunosuppressive therapy, but long-term systemic use of steroids has a considerable risk, putting a disease burden on patients.^[11]^ Therefore, it is particularly important to find more effective ways to treat or prevent the occurrence of adverse reactions.

In recent years, traditional Chinese medicine plays a more and more important role in the treatment of diseases. Some studies have shown that the combination of traditional Chinese and Western medicine has achieved obvious curative effect in the treatment of myasthenia gravis. The combination of traditional Chinese and Western medicine has obvious advantages over the simple treatment of Western medicine.^[12]^ Myasthenia gravis belongs to the category of “impotence syndrome” in traditional Chinese medicine. The basic pathogenesis is the loss of essence and qi of the 5 internal organs, loss of vital blood and body fluid, loss of tendon, unable to bind bones, and benefit joints.^[13]^ Buzhong Yiqi decoction contains traditional Chinese medicines such as Radix Astragali, Atractylodes macrocephala, and Codonopsis pilosula, which can strengthen the spleen, tonify the spleen and benefit the lung. Angelica sinensis, cohosh, Bupleurum, tangerine peel, and licorice can play the role of tonifying blood and invigorating blood, invigorating qi and invigorating spleen, raising Yang qi.^[14]^ Modern studies have shown that Buzhong Yiqi decoction can regulate humoral immune function through the interaction between drugs, inhibit the production of anti-acetylcholine receptor antibodies (AchR-Ab) and reduce the damage to muscle junctions, in order to improve the symptoms of myasthenia.^[15]^

However, the outcome indicators of different studies are different, and the data of some articles are incomplete or unable to extract data. The sample size of some studies is small or unknown, so there are some differences between different research results. The purpose of this study is to provide evidence-based basis for Buzhong Yiqi decoction combined with Western medicine in the treatment of myasthenia gravis through systematic review and meta-analysis of published randomized controlled trials.

## 2 Methods

### 2.1 Protocol register

This protocol of systematic review and meta-analysis has been drafted under the guidance of the preferred reporting items for systematic reviews and meta-analysis protocols (PRISMA-P). And, this research scheme has been registered with registration number on the OSF platform (Registration number: DOI 10.17605/OSF.IO/MXUPK).

### 2.2 Ethics

Since the study does not require the recruitment of patients with myasthenia gravis and the collection of patient information, it does not involve ethical issues. So, it does not need to be approved by the Ethics Committee.

### 2.3 Eligibility criteria

#### 2.3.1 Types of studies

We will collect all randomized controlled trials of Buzhong Yiqi decoction combined with Western medicine in patients with myasthenia gravis, regardless of their blindness, publication status or location, conducted only in Chinese and English.

#### 2.3.2 Research object

Patients with a clear diagnosis of myasthenia gravis, excluding patients with MG caused by congenital, family inheritance, drugs, etc., as well as patients with infectious diseases or severe organic diseases. However, the nationality, race, sex, age, occupation, course of disease, and onset time of the patient are not limited.

#### 2.3.3 Intervention measures

The control group will be treated with routine Western medicine, including cholinesterase inhibitor, glucocorticoid, immunosuppressant, and so on. The treatment group will be treated with Buzhong Yiqi decoction or its modified prescription on the basis of treatment in the control group, which had no restriction on the dosage form, dosage and course of treatment of Buzhong Yiqi decoction.

#### 2.3.4 Outcome index

1. Quantitive MG score (QMG).
2. Detection of AchR-Ab.
3. Detection of the number of Tregs cells.
4. Total efficiency.
5. Recurrence rate.
6. The incidence of adverse reactions.

### 2.4 Exclusion criteria

1. Articles that are inconsistent with the outcome indicators of this study.
2. Articles for which the full text is not available and the author of the communication is still unable to obtain the relevant data and information.
3. Articles in which the data is missing and the contact author is still unable to obtain complete data.
4. Non-clinical randomized controlled trials, such as self-control, retrospective studies, reviews, etc.
5. Study on the treatment group or control group combined with other traditional Chinese medicine, acupuncture, massage, and other traditional Chinese medicine treatment.

### 2.5 Retrieval strategy

Use “Buzhong Yiqi decoction,” “Western medicine,” “myasthenia gravis”, and other items as Chinese search words in Chinese databases such as China Science and Technology Journal Database, China National Knowledge Infrastructure, China Biomedical Database, Wanfang data knowledge Service platform, and so on. Take “medicine,” “Myasthenia Gravis,” “myasthenia reaction,” and “bulbospinal paralysis” as English search words, search them in Embase, PubMED, the Cochrane Library, Web of Science, and other English databases, and search them manually on Google academic, SCI-HUB, Baidu academic, and Lantern. The search time is from the establishment of the database to December 2020, and the domestic and foreign literatures of Buzhong Yiqi decoction combined with Western medicine in the treatment of myasthenia gravis will be collected. Take PubMed as an example, the retrieval strategy is shown in Table 1.

**Table 1:** PubMed database search strategy.

Number	Search terms
#1	buzhong yiqi decoction [Title/Abstract]
#2	Buzhong Yiqi Pill[Title/Abstract]
#3	BZYQT[Title/Abstract]
#4	Bu Zhong Yi Qi Wan[Title/Abstract]
#5	#2 OR #3 OR #4
#6	Myasthenia Gravis [MeSH]
#7	Myasthenia Gravis [Title/Abstract]
#8	bulbospinal paralysis [Title/Abstract]
#9	myasthenia reaction [Title/Abstract]
#10	Myasthenic Syndromes [Title/Abstract]
#11	#6 OR #7 OR #8 OR #9 OR #10
#12	#5 AND #11

### 2.6 Data screening and extraction

According to the methods of study selection in Cochrane intervention systematic Evaluation Manual 5.0, the 2 researchers introduce the literature into EndNote X7 according to the inclusion and exclusion criteria, then set up a group, check the repetition, screen the literature that obviously do not meet the requirements by reading topics and abstracts, and then screen the literature that meet the requirements again. It is difficult to determine whether it is included or not because of the differences and the third researcher discusses and determines whether it will be included or not. At the same time, Excel 2013 will be used to extract relevant information, including

1. the first author of the literature, the year of publication;
2. the basic situation of the subjects: physique, course of disease, age, height, weight, course of treatment, sex, sample size;
3. the intervention methods of the treatment group and the control group: the treatment group will be treated with Buzhong Yiqi decoction combined with Western medicine, and the control group will be treated with routine Western medicine or operation;
4. outcome index: Quantitive MG score, Tregs cell quantity detection, AchR-Ab content detection;
5. quality evaluation elements of the literature;
6. incidence of adverse reactions;
7. methodological information of the literature.

**Figure 1:**
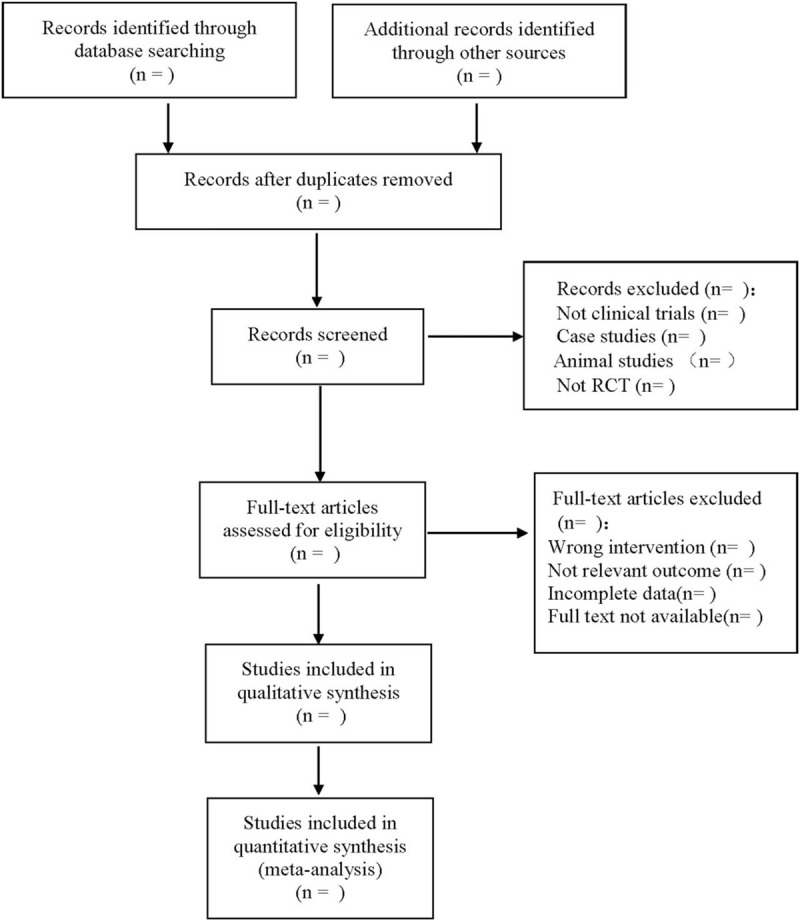
The process of literature screening.

The PRISMA flowchart is used to show the research selection process (Fig. 1).

### 2.7 Literature quality evaluation

The Cochrane collaboration's tool for assessing risk of bias, a risk bias assessment tool built into Review Manager 5.3 software, will be used to evaluate the risk bias of the included study. According to the performance of the above evaluation items in random sequence generation, allocation concealment, blinding of participants and personnel, blinding of outcome assessment, incomplete outcome data, selective reporting and other bias, the 2 researchers give low-risk, unclear, and high-risk judgments item by item, and cross-check them respectively after completion. If there are differences, they need to discuss, and if they cannot reach an agreement, they will agree with the third party researchers.

### 2.8 Statistical analysis

RevMan5.3 software will be used for meta-analysis. The second classification variable uses relative ratio to represent; for continuous outcomes, if the measurement tool and unit of measurement are consistent, the weighted mean difference is used to express. If the measurement tool or unit of measurement is inconsistent, the standard mean difference is used as the effect quantity, all expressed as 95% confidence interval. Heterogeneity will be determined by χ^2^ and *I*^*2*^ values, if *P ≥* .1*, I*^*2*^*≤* 50%, it indicates that the heterogeneity is low, and the fixed effect model is used for meta-analysis. If *P < *.1, *I*^*2*^ > 50%, the source of heterogeneity should be analyzed because there is heterogeneity between studies. Subgroup analysis will be used to deal with clinical heterogeneity. If there is no obvious clinical heterogeneity and methodological heterogeneity, statistical heterogeneity is considered, so the random effect model is used for analysis. If the clinical heterogeneity is too obvious to perform subgroup analysis, there is no meta-analysis, only descriptive analysis.

#### 2.8.1 Dealing with missing data

If there are missing data in the article, contact the newsletter author or the first author through the email to get the complete data. If the author has lost the relevant data, or is unable to contact the author so that the complete data cannot be obtained, a descriptive analysis is carried out and no meta-analysis is carried out.

#### 2.8.2 Subgroup analysis

The subgroup analysis will be carried out according to the severity of the patients, and the subgroup analysis will be carried out according to the type of Western medicine. The subgroup analysis will be carried out according to the course of Buzhong Yiqi decoction.

#### 2.8.3 Sensitivity analysis

In order to judge the stability of the outcome index, sensitivity analysis is used to analyze each outcome index.

#### 2.8.4 Assessment of reporting biases

If the number of studies included in an outcome indicator is not less than 10, a funnel chart is used to assess publication bias. In addition, Egger and Begg test will be used for the evaluation of potential publication bias.

#### 2.8.5 Evidence quality evaluation

The quality of evidence and the level of recommendation are evaluated according to the international GRADE system. According to the effect of Buzhong Yiqi decoction combined with Western medicine in the treatment of myasthenia gravis, the credibility of the evaluation results will be divided into high quality, medium quality, low quality, and very low quality.

## 3 Discussion

Myasthenia gravis is an autoimmune neuromuscular connectivity disorder mediated by antibodies against postsynaptic membrane-related proteins at neuromuscular junctions.^[16,17]^ Myasthenia gravis (MG)-related myasthenia gravis limits the daily function of patients. MG patients often have mental disorders, fatigue, decreased self-efficacy, and other subjective symptoms, which affect their lives.^[18]^ Thymus plays an important role in the pathogenesis of MG, 70% of patients with thymic follicular hyperplasia, and 20% of patients with thymoma.^[19]^ Activated T cells, B cells, and plasma cells play an important role in the production of pathogenic autoantibodies against MG and the induction of neuromuscular junction inflammation. Chronic inflammation mediated by helper T cell 17 (Th17) cells, follicular Th cells promoting the production of autoantibodies by B cells and plasma cells, regulatory T (Treg) cell dysfunction activating immune response may be the causes of exacerbation of MG.^[20]^ Although the standard treatment of myasthenia gravis includes steroids, acetylcholinesterase, rituximab, immunosuppressants and thymectomy, the number of relapses, and myasthenia gravis crises is still increasing.^[21]^

In recent years, traditional Chinese medicine combined with Western medicine has achieved good results in the treatment of myasthenia gravis.^[22]^ Buzhong Yiqi decoction is the main selection of traditional Chinese medicine in clinic. Studies have shown that Astragalus membranaceus and Atractylodes macrocephala contains polysaccharides, which can promote DNA synthesis, improve blood microcirculation and protect liver and analgesia. Atractylodes macrocephala polysaccharide is an important immunomodulator, which can regulate gastrointestinal function, enhance immunity and protect liver. Chinese wolfberry is rich in vitamins and trace elements, which can improve the phagocytosis of macrophages and enhance non-specific immune function. Angelica is rich in vitamins and amino acids, which can effectively eliminate excess free radicals, increase blood flow, and promote blood circulation. it has anti-inflammatory effect. Bupleurum can promote the production of antibodies, and the whole prescription can effectively improve the immune function of patients, promote blood circulation and improve the clinical effect.^[15,24]^ To sum up, Buzhong Yiqi decoction combined with Western medicine can effectively relieve the clinical symptoms of elderly patients with MG, regulate the number of Treg cells and Th17 cells, improve the level of serum AchR-Ab, improve immune function, and clinical efficacy, which is worthy of clinical application.^[25]^

However, due to the biased publication of the literature, different clinical absolute scoring standards, less literature inclusion, only computer retrieval, no manual retrieval, and other reasons, a comprehensive quantitative analysis cannot be carried out. Therefore, in the future, we should strictly according to the requirements of evidence-based medicine, use large samples, high-quality randomized controlled rials, to verify the efficacy, and safety of Buzhong Yiqi decoction combined with Western medicine in the treatment of myasthenia gravis.

## Author contributions

**Data curation:** Gang Zong, Shiliang Liu.

**Funding acquisition:** Yulei Hu.

**Literature retrieval:** Shiliang Liu and Zunling Chen

**Software:** Gang Zong.

**Supervision:** Zunling Chen.

**Writing – original draft:** Gang Zong, Shiliang Liu.

**Writing – review & editing:** Gang Zong, Yulei Hu.
